# Experimental Researches on the Action of Oils on the Animal Economy

**Published:** 1845-06

**Authors:** 


*Experimental Researches on the Action of Oils on the Animal Economy.* By MM. Gluge and Thiernesse.—Olive oil and cod-liver oil were the two fatty oils used by MM. Gluge and Thiernesse in their experiments, and they were administered to dogs, rabbits, and kids, by injection into the veins, and also by the mouth. When either oil was injected into the veins, it usually produced dyspnœa, debility, painful sensation, and some affection of the circulation. The animals, after a few days, usually recovered from the first injection, but died very shortly after repeating the operation, from difficulty of breathing, rapidly failing pulse, and complete prostration. On dissection the lungs, liver, and kidneys were usually found in a more or less morbid state, exhibiting symptoms of inflammatory action; globules of oil, and a peculiar kind of crystals were also very commonly noted in their structures. The blood was more or less fluid, and peculiar crystals frequently remarked. When pure cod-liver oil was used, its action did not seem to differ from that of olive oil; but the brown or dark-colored cod-liver oil produced rapid death, often within an hour after its injection. In these cases the lungs were gorged with black blood, and emphysematous; and the blood, besides being fluid, contained a much larger proportion of oily globules, and of the peculiar crystals above alluded to.

When the oil was given by the mouth the dose was daily increased from a table spoonful. For several days or weeks the animal maintained its health, but at length it began to refuse nourishment, got feverish, suffered from dyspnœa, agitation of the limbs, weakness, and gradually sunk. In every case the lungs were found inflamed, congested, often hepatized, and everywhere penetrated with oil. The liver and kidneys were also in general more or less engorged with blood.

From these experiments the authors deduce the following conclusion:

1. That olive oil and pure fish oil do not act differently on the animal economy, whether taken by the mouth or injected into the veins, excepting in a few exceptional cases in dogs, when the fibrin of the blood and the muscles seem to acquire additional density when cod-liver oil was taken internally. But this result was not constant.

2. That unclarified dark-coloured fish oil, when injected into the veins produces rapid asphyxia and decomposition of the blood, as evidenced by the irregular form of the blood-globules, and the presence of crystals in that fluid.

3. That the fatty oils, however introduced into the body, have a natural tendency to deposit themselves in the lungs, liver, and kidney.

4. That in these organs they are deposited in two different ways; they exude into the parenchyma through the capillary blood-vessels, or they become deposited in the biliary cells, the pulmonary vesicles, or the uriniferous canals.

5. That if a small quantity of oil be injected at once, the animal may recover from the effects.

6. That the effects of oil administered by the mouth varies much according to the dose, and the period of time when the animal takes it.

7. That when the dose is daily increased, the animals lose their appetite, become lean, cough, suffer from dyspnœa, and at last present all the signs of a violent pneumonia, to which they fall victims.

8. That the pathological appearances consist of total or partial hepatization of the lungs, with accumulation of fatty matters in the parenchyma of the lungs, liver, and kidneys.

9. That the hepatization of the lungs is always, as to its extent, in proportion to the quantity of oil introduced into the system by the stomach.

10. That the oil administered by the mouth is absorbed by the intestinal villi, and being thrown by the circulation on the lungs, liver, and kidneys, produces fatty degeneration of these organs.

11. That the particular-form of pneumonia, caused by large doses of oil, had been described by the older physicians, under the name of bilious pneumonia, and had not been recognised by the moderns.

12. That when oil is given medicinally for any length of time, we ought to exercise the muscles as well as the lungs, and watch carefully its action, for although cod-liver oil may be a therapeutic agent of much power, it is also one whose use is not unattended with danger, as commonly administered.—*Edingburgh Med. and Surg. Journ., from Gazette Medicale de Paris*, 9th Nov. 1844.

